# Bismuth-Based Halide Perovskites for Photocatalytic H_2_ Evolution Application

**DOI:** 10.3390/molecules28010339

**Published:** 2023-01-01

**Authors:** Costanza Tedesco, Lorenzo Malavasi

**Affiliations:** Department of Chemistry and INSTM, University of Pavia, Via Taramelli 16, 27100 Milan, Italy

**Keywords:** metal halide perovskites, photocatalysis, hydrogen generation

## Abstract

Metal halide perovskites (MHPs), in particular lead-based perovskites, have earned recognized fame in several fields for their outstanding optoelectronic properties, including direct generation of free charge carriers, optimal ambipolar charge carrier transport properties, high absorption coefficient, point-defect tolerance, and compositional versatility. Nowadays, this class of materials represents a real and promising alternative to silicon for photovoltaic technologies. This worthy success led to a growing interest in the exploration of MHPs in other hot research fields, such as solar-driven photocatalytic water splitting towards hydrogen production. Nevertheless, many of these perovskites show air and moisture instability problems that considerably hinder their practical application for photocatalytic water splitting. Moreover, if chemical instability is a problem that can be in part mitigated by the optimization of the chemical composition and crystal structure, the presence of lead represents a real problem for the practical application of MHPs in commercial devices due to environmental and healthcare issues. To successfully overcome these problems, lead-free metal halide perovskites (LFMHPs) have gained increasing interest thanks to their optoelectronic properties, comparable to lead-based materials, and their more eco-friendly nature. Among all the lead-free perovskite alternatives, this mini-review considers bismuth-based perovskites and perovskite derivatives with a specific focus on solar-driven photocatalysis application for H_2_ evolution. Special attention is dedicated to the structure and composition of the different materials and to the advantage of heterojunction engineering and the relative impact on the photocatalytic process.

## 1. Introduction

### 1.1. Metal Halide Perovskite Photocatalysts

The World Energy crisis and the environmental pollution that has led to the well-known climate change are the main problems faced by scientists nowadays. In such a scenario, the development of renewable and green energy has become inevitable. Considering that solar energy is continuous and inexhaustible and it strikes the earth’s surface constantly, the effective utilization of this source of energy is one of the main goals to succeed in alleviating the energy crisis and the wicked consequences of the excessive use of fossil fuels of the last century [1,2,3]. In nature, it is possible to observe the perfect example of the conversion of sunlight into fuels; the photosynthetic processes that occur in plants are the exact representation of the storing of the energy from the incident solar irradiation in the form of the chemical bond [4,5,6]. On this path, looking for an efficient semiconductor material that can emulate the natural conversion of sunlight into fuels that take place in the leave’s plants is the main focus of the solar-driven photocatalysis research field. Metal halide perovskites (MHPs) with general formula ABX_3_ (A is a monovalent organic o inorganic cation such as methylammonium MA, formamidinium FA, Cs etc.; B is a bivalent metal such as Pb, Sn, Ge, and X is the halide anion, Cl, Br, and I) represent a revolutionary class of materials for photovoltaics and optoelectronics fields thanks to their outstanding and scalable optoelectronic properties as well as their low cost, and ease of synthesis [7,8]. Traditionally, most of the best-performing photovoltaic materials in recent years have been applied to photocatalytic hydrogen production processes, and the same happened to the metal halide perovskite materials due to the tunability of their electron band structure which can place the band edges in good positions to perform photocatalytic reactions [2,3,4,5,9,10,11,12,13]. Although Pb-based perovskites have shown interesting achievements, the substitution of toxic Pb, well-known to be harmful to human health and the environment, has gradually but rapidly become another challenge [9,10,11,12,13]. The Pb presence is already an obstacle to envisioning large-scale production and distribution of perovskite-based photovoltaic devices but has become even more pressing for photocatalytic applications [2,9,10,11,12,13,14,15]. Lead toxicity is an important environmental disease, and its effects on the human body are devastating; there is almost no function in the human body that is not affected by lead toxicity. Lead metal bears unique physical and chemical properties that make it suitable for a large number of applications for which humans have exploited its benefits from historical times, and thus it has become a common environmental pollutant [16]. The interest in lead-free perovskites is born to overcome the Pb presence, but nowadays, we can affirm that the Pb-free perovskites represent a real and promising class of semiconductors materials standalone. The bivalent Pb cation can be replaced by other metal ions with lower toxicity, such as divalent Sn^2+^ or Ge^2+^, trivalent Bi^3+^ or Sb^3+^, and tetravalent Sn^4+^ or Ge^4+^. Unfortunately, Sn^2+^ and Ge^2+^ ions are extremely sensitive to air and moisture and easily oxidated to Sn^4+^ and Ge^4+^, respectively, which currently hinders their practical application in semiconductor materials for water splitting [4,5,14]. 

### 1.2. Lead-Free Metal Halide Photocatalysts

**Figure 1:**
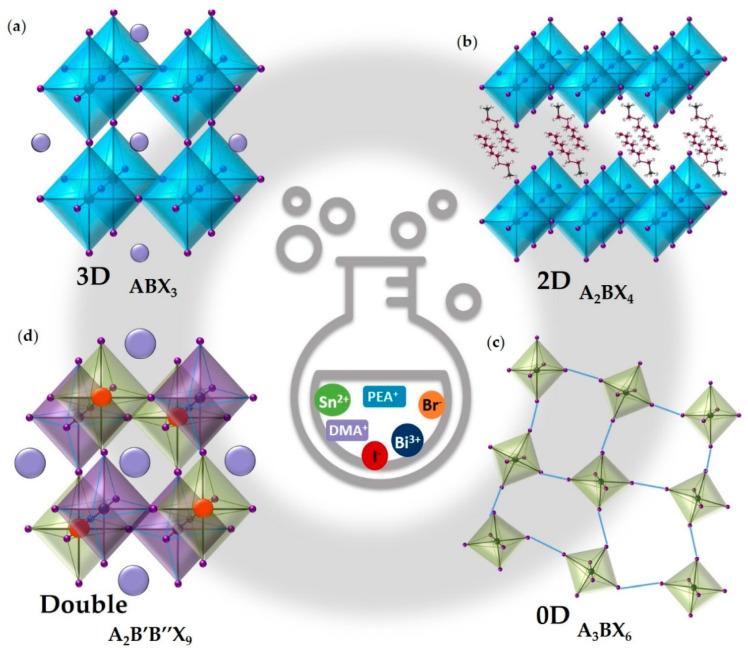
Listorti et al. reported complete examples of schematic crystal structures with the related chemical formula of (**a**) 3D, (**b**) 2D, (**c**) 3D, and (**d**) double metal halide perovskites used for H_2_ evolution [19].

A promising way to overcome the instability problem faced by the Ge- and Sn-based perovskites is the replacement of two Pb^2+^ cations with one tetravalent cation (or one monovalent and one trivalent cation) to form the double perovskite with formula A_2_B(IV)X_6_ or otherwise substitute three Pb^2+^ cations with two trivalent cations to form A_3_B(III)_2_X_9_ stoichiometry. Figure 1 reported the main examples of crystal structures with the related chemical formula [4]. Beyond the classic stoichiometry for the 2D perovskites, 2D perovskite can also show AB_2_X_5_ and A_3_B_2_X_9_ stoichiometries consisting of the alternation of A^+^ ions and [B_2_X_5_]^−^ polyhedra and [B_2_X_9_]^3−^ isolated clusters, respectively. In order to maintain charge neutrality, it can be easily understood that ordered metallic vacancies will be formed, resulting in a reduced electronic dimensionality (0D or 2D). Among all the possible substitutes for Pb cations, the Bi^3+^ isoelectronic with Pb^2+^ adopts a similar valence shell electron lone pair 6s^2^ and has a nearly equivalent effective ionic radius to Pb^2+^ and for these reasons, the interest in these types of Bi-based perovskites is still growing. In the most crystal structure of Bi-based perovskite materials, there are corner-sharing BiX_6_ octahedra located at the surface of perovskite crystals, and trivalent cations generally form A_3_M_2_▢X_9_ (▢ is the vacancy) structure with 2/3 occupancy of the M sites A_3_M_2_▢X_9_ perovskite. Therefore, Bi-based halide perovskites have gained special attention in very recent years. On the other hand, the trivalent cations Bi^3+^ are not able to form a continuous 3D structure and lead to the formation of derivatives with low dimensionality [4,6,17,18]. These halide perovskites Bi-based derivatives can show, in many cases, an indirect band gap that leads to poor photovoltaic performance but may be of interest for the photocatalytic production of H_2_ in an aqueous solution [3,4,5]. Based on these considerations, this mini-review will be focused on the research efforts exploiting the use of Bi-based perovskite and perovskite derivatives for the H_2_ generation by showing recent outstanding achievements as well as their main limits and reporting promising future perspectives. Before considering the different classes of Bi-based materials for hydrogen production, we will briefly describe in the next sections the main strategies used to date to exploit the use of MHPs in H_2_ generation with also reference to the underlying mechanisms. 

### 1.3. Design and Mechanism of H_2_ Production by Metal Halide Photocatalysts

**Figure 2:**
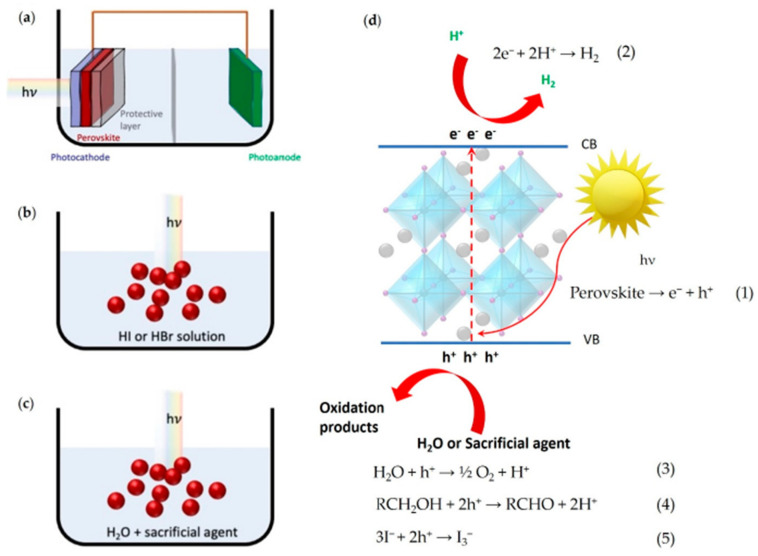
Solar-driven perovskite-based H_2_ production system: (**a**) photoelectrochemical (PEC) cell; (**b**) particulate photocatalyst system in dynamic equilibrium with the corresponding halogen acid; (**c**) particulate water-stable photocatalyst system. (**d**) Schematic representation of the processes on the perovskite photocatalyst surface under irradiation and possible reactions involved in the different systems [19].

The photocatalytic processes mentioned, which occur under visible-light irradiation, often require the use of a water environment, and this represents a serious obstacle for metal halide perovskites utilization due to their intrinsic instability in aqueous media. Air (oxygen and moisture), illumination, and polar solvent can rapidly decompose MHPs, and, in order to improve the stability of these materials, the photocatalytic reactions were often performed in a halogen acids medium where halide perovskites can reach a dissolution-recrystallization equilibrium. In addition, the substitution of organic cations (MA and FA) with all inorganic ions showed an interesting increase in stability. Obviously, the collective goal should be performing H_2_ generation from pure water splitting, but, unfortunately, very few examples of water-stable metal halide perovskite have been reported to date. In Figure 2, Listorti et al. reported the solar-driven perovskite-based systems showing the different mediums exploited to generate H_2_ [4,6]. Tree main paths have successfully been exploited to overcome the issue of the water instability of these materials; the first one (Figure 2a) foresees the encapsulation of the active layer of perovskite, avoiding water contact. This approach allowed the inclusion, for the first time, of MHPs as photocathodes in complete photoelectrochemical (PEC) cells. The second strategy (Figure 2b) exploits complex dynamic equilibria between perovskite precursors and perovskite powders dispersed in solution. The last strategy implies the use of water-stable MHPs (Figure 1c). 

**Figure 3:**
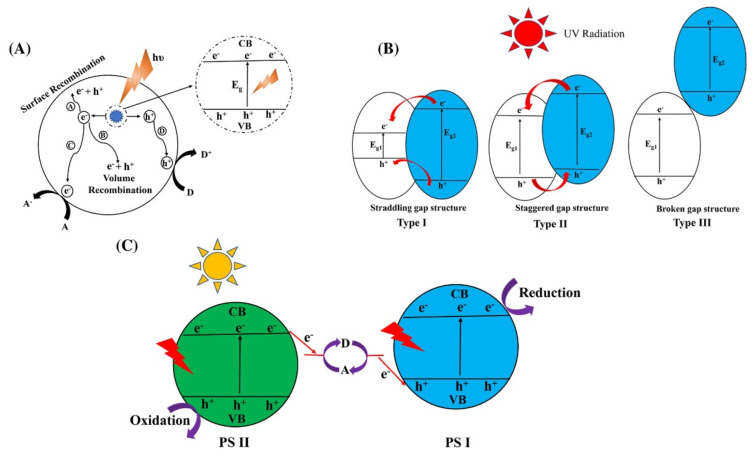
(**A**) Schematic illustration showing the electron-hole pathways in a photocatalytic process. (**B**) Schematic illustration showing three different types of semiconductor heterojunctions for photo-generated charge separation. (**C**) Schematic illustration of a Z-scheme photocatalytic system under light illumination [6,28,29]. *Source A* reprinted with permission from ref [28]: Copyright 1995 American Chemical Society [28]. *B, C* reprinted with permission from ref [29]: Copyright 2017, Jhon Wiley & Sons., Inc. [29].

Considering the intrinsic water instability of metal halide perovskites and the rapid recombination of the charge carries, during these last years, several strategies have been applied to solve these problems, and one of the most explored has been the construction of heterojunctions to boost the extraction of photogenerated electrons and holes [20,21,22,23,24,25,26,27]. In Figure 3, it is reported a schematic illustration showing the electron-hole pathways in a photocatalytic process (Figure 3A) and the different types of heterojunctions that can be devised through the coupling of two semiconductors (Figure 3B). A conventional Z-scheme photocatalytic system (Figure 3C) is composed of two different semiconductors, photocatalyst I (PS I) and photocatalyst II (PS II), and an acceptor/donor (A/D) pair. During the photocatalytic reaction, since PS I and PS II are not in physical contact, photogenerated electrons migrate from the CB of the PS II to the VB of the PS I through an A/D pair reaction, where A is reduced into D by reacting with the photogenerated electrons form the CB of the PS II. After that, the D is oxidized into A by the photogenerated holes from the VB. Since a single semiconductor rarely meets all the stringent requirements for an efficient photocatalytic reaction, a second phase is specifically designed to couple to the semiconductor, which will form an effective heterojunction or provide support to the main semiconductor. Compared to the single semiconductor, the heterojunction experiences higher light adsorption ability, more efficient charge separation and transfer as well as better stability [6]. To improve the photocatalytic performances, halide perovskites were included in heterojunction with various common and efficient semiconductor materials such as graphene oxide (GO), g-C_3_N_4_, TiO_2_, metal-organic frameworks (MOFs) [4,6,28]. 

After having assessed the main strategies used to exploit the use of MHPs in hydrogen production, we will now review in more detail the l Bi-based halide perovskite applied for photocatalytic H_2_ evolution. The following sections are organized by considering the hybrid systems, namely containing one organic cation and the fully inorganic Bi-based perovskites and perovskites derivatives. For both classes of materials, we further highlighted the structural motif characterizing the different phases, i.e., 0D, 2D, or 3D, according to the commonly used notation in the field. For all the compositions reported, we will give an overview of the strategy used to promote H_2_ production, providing information on the proposed mechanism, and highlighting the environment where the photogeneration of hydrogen is carried out since this aspect determines the experimental setup and the materials and device engineering.

## 2. Hybrid Bi-Based Halide Perovskites

### 2D and 2D Bi-Based Halide Perovskites

**Figure 4:**
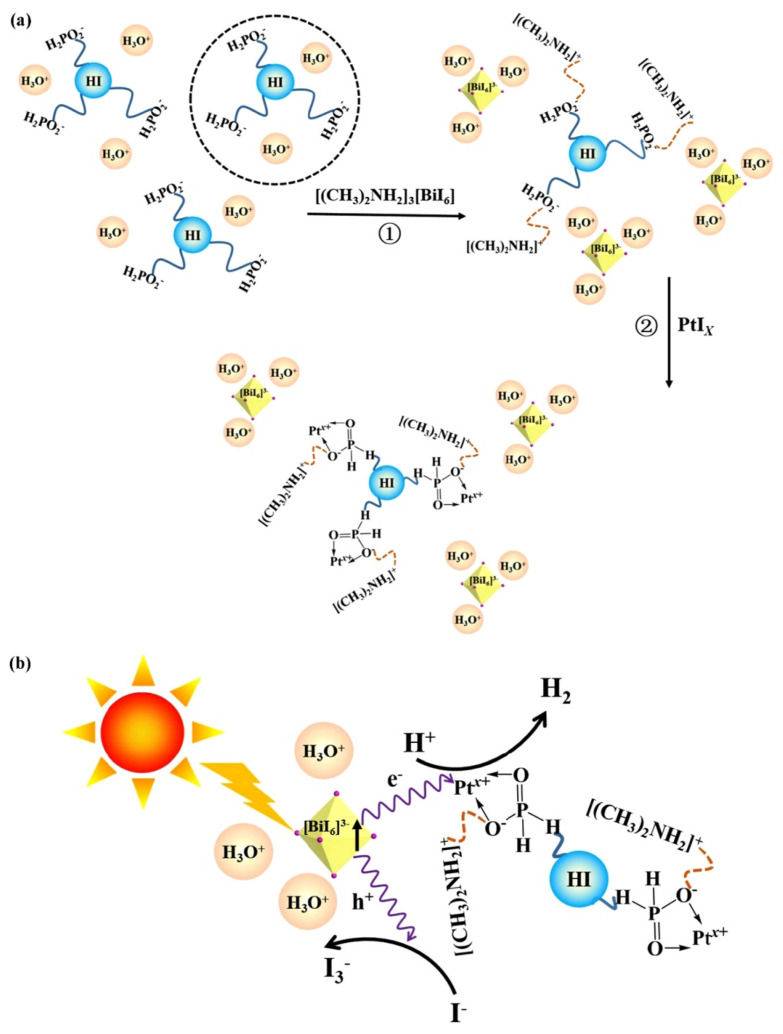
Schematic illustration of (**a**) the conversion process in the colloidal solution and (**b**) the proposed mechanism for photocatalytic hydrogen generation [29].

In 2018 Zhao et Al. reported for the first time a Bi-based 0D perovskite for H_2_ production exploiting a solvothermal method [30]. One of the main challenges is still developing photocatalysts with high efficiency in a wide range of visible light, and the obtained organic-inorganic hybrid PtI_x_/[(CH_3_)_2_NH_2_]_3_[BiI_6_] for photocatalytic hydrogen production from hydroiodic acid harvested a wide range of visible light (up to 630 nm) proving to be a highly active system stable for more than 100 h. For the first time, they succeeded in the use of a hybrid Bi-based perovskite as a photocatalyst in a well-dispersed system for hydrogen evolution. Moreover, they showed a facile solvothermal method to synthesize a lead-free organic-inorganic hybrid perovskite, followed by decoration with platinum, which forms well-dispersed systems in the HI-H_3_PO_2_ solution for H_2_ generation. The authors attributed the superior photocatalytic performance to the collision between [BiI_6_]^3−^ and Pt ions (Pt^2+^ and Pt^4+^) since this mechanism could be responsible for efficient charge separation. The photocatalytic H_2_ evolution process was evaluated in HI solution, and due to the possible interference of I^3−^ ions (generated during photocatalytic HI splitting), H_3_PO_2_ was added to the reaction solution. The introduction of [(CH_3_)_2_NH_2_]_3_[BiI_6_] powders in the HI-H_3_PO_2_ solution did not show any precipitation demonstrating outstanding stability even after 200 days, as demonstrated by XRD measurements before and after the photocatalytic process. To obtain the highest photocatalytic activity of [(CH_3_)_2_NH_2_]_3_[BiI_6_], the optimization of the amount of the photocatalyst was performed under illumination around 425 nm. The authors showed that the optimal rate of H_2_ evolution was achieved with a concentration of photocatalyst of 20 mg/mL, and since it is well known that Pt is a good co-catalyst for H_2_ production in the photocatalytic process, Pt was introduced in order to improve the performances of their system at varying amounts from 0 to 1 wt%. The photocatalytic activities of Pt/[(CH_3_)_2_NH_2_]_3_[BiI_6_] showed a dramatic enhancement after Pt loading, reaching an H_2_ production of 186.5 μmol g^−1^ for the sample with 1% of Pt, which is 12.3 times higher than that of the non-loaded [(CH_3_)_2_NH_2_]_3_[BiI_6_]. Inspired by these results, the authors proposed the possible photocatalytic mechanism in the present system in Figure 4. While the photocatalytic mechanism shown does not seem to be the traditional one that takes place in heterojunctions semiconductors applied for H_2_ photogeneration, this paper represents a milestone for future effective application of lead-free Bi-based perovskite in photocatalysis [3,30]. 

In 2019 Guo et Al. obtained the environmentally friendly hybrid lead-free perovskite MA_3_Bi_2_I_9_ (MA = methylammonium) by employing a facile solvothermal method [31]. The authors proved the excellent phase stability of MA_3_Bi_2_I_9_ in hydriodic acid aqueous solution with different concentrations under visible light irradiation, with this phase showing satisfactory cycle stability after 70 h of repeated H_2_ evolution without any degradation. With the addition of Pt as a cocatalyst, the photocatalytic rate for H_2_ evolution was about 169.21 µmol g^−1^ h^−1^, resulting 14 times improved compared with the bare perovskite and with a solar chemical conversion efficiency of 0.48%. In order to investigate the phase stability of MA_3_Bi_2_I_9_, different volume ratios of hydroiodic acid and deionized water were mixed to prepare HI aqueous solutions, and from the XRD diffraction patterns, the author demonstrated that MA_3_Bi_2_I_9_ maintained a satisfactory phase stability when transferred in each solution with different concentrations of HI. This result differed from the previously reported phase conversion of MAPbI_3_, and the improved stability of MA_3_Bi_2_I_9_ could be attributed to the oxidation state of bismuth (III) [31]. The MA_3_Bi_2_I_9_ powder exhibited a bright red color with a high absorbance in visible light leading to a large number of absorbed solar photons. Under visible-light irradiation (λ ≥ 400 nm), the photogenerated electrons in MA_3_Bi_2_I_9_ were excited into the conduction band, separated from the photogenerated holes, providing the path for H^+^ reduction to H_2_, and at the same time, the photogenerated holes in the valence band were used to oxidize I^−^ to I^3−^. However, the authors needed to add hypophosphorous acid (H_3_PO_2_) to the saturated solution systems because the oxidation product I^3−^ anion could interfere with the light absorption of the perovskite powder, as was already previously experienced. The hypophosphorous acid had the role of selective reducing agent for I^3−^ to achieve stable long-term H_2_ evolution without interfering with the UV–vis absorption spectrum, confirming its crucial role in the reduction of I^3−^ ions. Among the MA_3_Bi_2_I_9_/Pt samples loaded with different amounts of Pt, the results from the MA_3_Bi_2_I_9_/Pt with 40 mg of MA_3_Bi_2_I_9_ and 2 mg of H_2_PtCl_6_·6H_2_O exhibited the most favorable hydrogen generation, showing about 169.21 µmol g^−1^ h^−1^ H_2_ production rate. These systems maintained a good crystallinity even after the photocatalytic HI splitting reaction, which resulted in being 14 times enhanced compared to the pristine MA_3_Bi_2_I_9_ [31].

**Figure 5:**
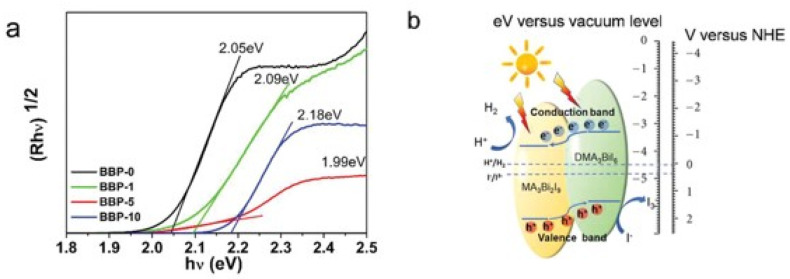
(**a**) Indirect-bandgap Tauc plot for all as-prepared samples. (**b**) Energy level diagram of the BBP-5 with a heterojunction of MA_3_Bi_2_I_9_ and DMA_3_BiI_6_ for the photocatalytic HI splitting. Reprinted with permission from ref [32]: Copyright 2020, Wiley-VCH GmbH [31].

In 2020 Tang et al. reported a successful in situ growth of heterojunctions developed at the interface of MA_3_Bi_2_I_9_ and tri(dimethylammonium)hexa-iodobismuthate (DMA_3_BiI_6_) by a facile solvent engineering technique [32]. They confirmed that air-stable MA_3_Bi_2_I_9_/DMA_3_BiI_6_ perovskite heterojunctions can reinforce the efficiency of photogenerated carrier separation and transport, eventually enhancing solar HI splitting efficiency in the absence of noble metal co-catalysts. The authors prepared several samples exploiting a solvothermal synthesis where isopropanol (IPA) was employed as a solvent with DMF added as a co-solvent. Ultraviolet–visible diffuse reflectance spectroscopy and XPS valence band spectroscopy were carried out to construct the band structure of all the prepared powder samples, labeled as BBP-x (bismuth-based perovskites with x = 0, 1, 5, and 10 volume percent of DMF in IPA). All the samples exhibited visible light absorption ranging from 300 to 650 nm. Through a Tauc plot (Figure 5a), they determined the optical bandgap energy of all the powder samples showing that those values were suitable for visible light-induced photocatalytic reaction, and from 5b, it is possible to observe the results for the energy level diagram of the BBP-5 with a heterojunction of MA_3_Bi_2_I_9_ and DMA_3_BiI_9_ for the HI splitting showing an efficient Type II heterojunction [32].

The driving force for the separation of photogenerated charge carriers could, therefore, be provided by the heterojunction band alignment of MA_3_Bi_2_I_9_ and DMA_3_BiI_6_ with the transfer of the photogenerated electrons from the conduction band of DMA_3_BiI_6_ to that of MA_3_Bi_2_I_9_, with the contemporary migration of the holes in the opposite direction (see Figure 4d). The BBP-5 perovskite with MA_3_Bi_2_I_9_/DMA_3_BiI_6_ heterojunctions showed high potential for hydrogen reduction and iodine oxidation as a result of enhanced spatial charge separation. It was confirmed that HI could efficiently be split into H_2_ and I^3−^ by all the precipitated Bi-based perovskite photocatalysts under visible light (λ ≥ 420 nm) illumination as a result of the suitable band structure, and among all the Bi-based perovskites samples, BBP-5 exhibited the best performance, providing a photocatalytic H_2_ evolution rate of 198.4 µmol h^−1^ g^−1^ without the addition of any metal co-catalysts. The outstanding results could be explained due to the relative positions of VB and CB of MA_3_Bi_2_I_9_ and DMA_3_BiI_6_ leading to a well-matched type II heterojunction and the consequent interfacial charge transfer pathway at the interface of MA_3_Bi_2_I_9_ and DMA_3_BiI_6_ [32]. 

**Figure 6:**
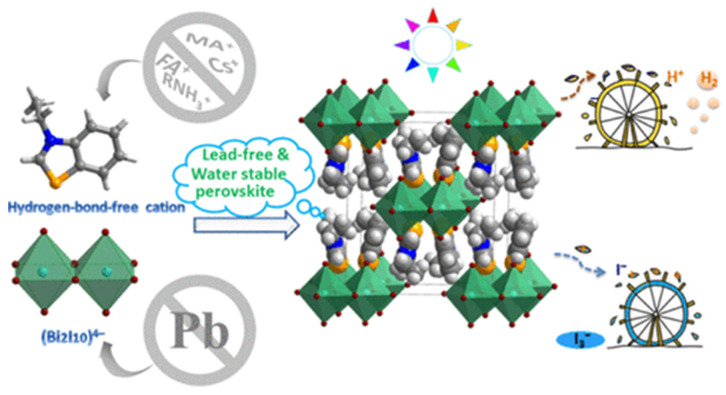
Zero-dimensional Bi-based perovskite (3-ethylbenzo[d]thiazol-3-ium)_4_Bi_2_I_10_ photocatalytic mechanism schematic representation. Reprinted with permission from ref [33]: Copyright 2020, American Chemical Society [33].

In 2020, Liu et al. synthesized a 0D Bi-based perovskite (EtbtBi_2_I_10_(Etbt=3-ethylbenzo[d]thiazol-3-ium) which contains dimeric (Bi_2_I_10_)^4−^ formed by edge-sharing (BiI_6_) octahedra being different from the binuclear cluster in the previously studied MA_3_Bi_2_I_9_. In this work, the authors demonstrated a hydrogen-bond-free strategy to synthesize moisture-stable hypo-toxic hybrid perovskite for photocatalytic application by replacing traditional protonated counter-cations with alkylated ones in a Pb-free hybrid system, which prevents water eroding hybrid perovskites via strong hydrogen bonds (see Figure 6) [33]. 

The photocatalytic performance of the powdery sample of EtbtBi_2_I_10_ was first evaluated under simulated sunlight irradiation, where the bare EtbtBi_2_I_10_ was used as the photocatalyst and evolved 6.8 μmol of H_2_ after 5 h. After loading 0.5 wt% Pt nanoparticles on EtbtBi_2_I_10_, the evolved H_2_ from HI and H_3_PO_2_ saturated solution was increased to 9.2 μmol, and this could be ascribed to the Pt that acts as the active sites for H_2_ evolution in a photocatalytic system, which greatly speeds up the reaction. Considering that HI cannot generate hydrogen alone, the evolved H_2_ is ascribed to the photocatalytic reactions on the EtbtBi_2_I_10_ semiconductor [33]. This novel system shows effective photocatalytic performance in HI splitting to generate hydrogen with performance comparable with MAPbI_3_. It is already known that the charge recombination on a hybrid perovskite semiconductor greatly decreases the photocatalytic performance, and in this work, a charge transportation modulation strategy was used with the aim of improving the photocatalytic performance of EtbtBi_2_I_10_. The hybridization strategy consists in introducing electron and/or hole transport pathways that could lead to a more effective photo-generated charge separation and transfer on the photocatalysts. The EtbtBi_2_I_10_ was hybridized first with the electron transfer agent TiO_2_ as well as SnO_2_ and Ta_2_O_5_, respectively, then Pt was loaded on the nanoparticles of TiO_2_ via a photo-reduction method and then hybridized with EtbtBi_2_I_10_ particles. After dispersion in HI saturated solution, the systems reached an H_2_ generation after 5 h of 59.9 µmol when adding Pt (1.25 wt%)/TiO_2_ nanoparticles to the perovskite. Finally, encouraged by the electron transportation promotion strategy, the rGO hole transportation channel was introduced into Pt/TiO_2_- EtbtBi_2_I_10_ system, showing that when the amount of rGO is 1.0 mg, the evolved H_2_ from the systems reached 83.8 µmol. Long-term stability is a vital quality for photocatalysts, and the cycling test for Pt/TiO_2_-EtbtBi_2_I_10_-rGO confirmed good system stability. Aside from showing a promising moisture-stable bismuth(III)-based hybrid perovskite for HER application, here the authors demonstrated an effective synthesis via water-stable and nontoxic hybrid perovskites for practical applications [33].

Concerning the intrinsic instability of the hybrid metal halide perovskite, in 2021, Zhao et al. reported an interesting strategy to stabilize DA_3_Bi_2_I_6_ in water using dimethylammonium iodide (DAI) without the assistance of acids or coatings. They proved that the perovskite remains stable for at least two weeks in water. Through the combination with Pt as cocatalyst in the form of PtCL_4_ to construct Pt-DA_3_BiI_6_ material, it was possible to achieve an HER of 5.7 µmol g^−1^ h^−1^ from HI and DAI solution, with an apparent quantum efficiency (AQE) of 0.83% at 535 nm. Even if the amount of hydrogen evolved is quite low, this paper could represent an inspiring work for achieving stabilized hybrid halide perovskite for H_2_ evolution from an aqueous solution [34].

## 3. Inorganic Bi-Based Halide Perovskite

### 2D and 3D Double Bi-Based Halide Perovskite

During 2020, it was possible to observe an intense growing interest in Bi-based perovskite systems for photocatalytic application, and Cheng et al. demonstrated an efficient co-precipitation method to obtain a series of full inorganic lead-free perovskites, namely Cs_3_Bi_2x_Sb_2-2x_I_9_ (x = 0.1, 0.3, 0.5, 0.7, 0.9) and determined their photocatalytic performance for hydrogen production in aqueous HI solution. Bi-based halide perovskites Cs_2_Bi_2_I_9_ (CBI) and its Sb-analogue (CSI) have been examined for photovoltaic application, but this was the first time that this class of perovskites was tested for hydrogen evolution reaction (HER). Through theoretical calculation, the authors showed how the introduction of Sb in the Cs_3_Bi_2x_Sb_2-2x_I_9_ (CBSI) solid solution could reduce the contribution of Bi metal ions to the conduction band, thus weakening the impact of defects and reducing the mid-gap states, quite common for phase containing Bi^3+^ cation [35]. The photocatalytic H_2_ evolution over CBI was evaluated in HI solution saturated with CBI and showed a quite low photocatalytic activity (1.12 µmol h^−1^) to further improve the performance, Cs_2_CO_3_ was added to the CBI HI solution to increase the Cs^+^ ions. Compared to the pristine solution, the addition of Cs_2_CO_3_ led to an increase in H_2_ evolution by a factor of 5. As it was mentioned above, the authors noticed that the doping of Sb in CBSI effectively abated the impact of Bi vacancy on the band structure, it was evaluated the photocatalytic activity of CBSI demonstrated that the H_2_ evolution activity of CBSI-0.3 (Cs_3_Bi_0.6_Sb_1.4_I_9_) was greatly improved, moreover with the adding of Cs_2_CO_3_, the amount of H_2_ evolution for the solution increased almost linearly with increasing the reaction time. At any given reaction period, CBSI-x had a higher H_2_ evolution activity compared with CBI or CSI, and the activity of the solid solution CBSI-x changed gradually as a function of x reaching the maximum at x ≈ 0.3 (78.6 µmol h^−1^). Moreover, CBSI-0.3 samples showed no significant decline in H_2_ evolution activity even after five consecutive cycles of the H_2_ evolution experiments (10 h for each cycle), and the apparent quantum efficiency of CBSI-0.3 was found to be 1.206% under irradiation at 420 nm light. The satisfactory system stability was confirmed by comparing the XRD patterns of CBSI-0.3 after being used for H_2_ evolution with the standard XRD pattern of CBI. In order to further improve the H_2_ evolution of CBSI-0.3, Pt nanoparticles were loaded as co-catalyst using a photo-reduction method during the photocatalytic process. The authors showed that the H_2_ evolution activity of CBSI-0.3/Pt is similar to that of the two best Pb-based photocatalysts for H_2_ evolution, and in the case of the Cs_3_Bi_0.6_Sb_1.4_I_9_ powder with Pt deposition, 463 µmol of H_2_ were generated after 5 h of light irradiance at 100 mW cm^−2^ with a solar HI splitting efficiency of 0.319%.

The photoelectrochemical properties of CBI, CSI, and CBSI-x were examined by using films, and the performance results indicated that much more photocarriers are accumulated on the surface of the CBSI-x films and participate in surface reduction reactions than on the surface of CBI or CSI proving that the doping represents a promising way to boost the photocatalytic performance [35]. 

Shortly thereafter, in 2021, Malavasi, Romani, and coworkers reported an experimental and computational study on the synergic coupling between Cs_3_Bi_2_Br_9_ perovskite derivative and g-C_3_N_4_. As we have previously shown, a few perovskites have been employed as heterogeneous photocatalysts for hydrogen evolution, but since most MHPs are unstable in an aqueous environment, the majority of experiments have been conducted in hydrohalic acids that prevent the complete dissolution of the perovskites by common-ion effect. However, the development of water-stable photocatalysts is becoming an imperative common goal to achieve a greener and more sustainable energy production road. On these bases, the authors of this paper attempted for the first time the application of a Bi-based perovskite for effective photocatalytic hydrogen evolution in aqueous media [36]. They reported the synthesis and characterization of a new heterogeneous photocatalyst, labeled Cs_3_Bi_2_Br_9_/g-C_3_N_4_, obtained by coupling the semiconductor Cs_3_Bi_2_Br_9_ perovskite to the already known photocatalyst g-C_3_N_4_, quite active under visible light irradiation [36].

**Figure 7:**
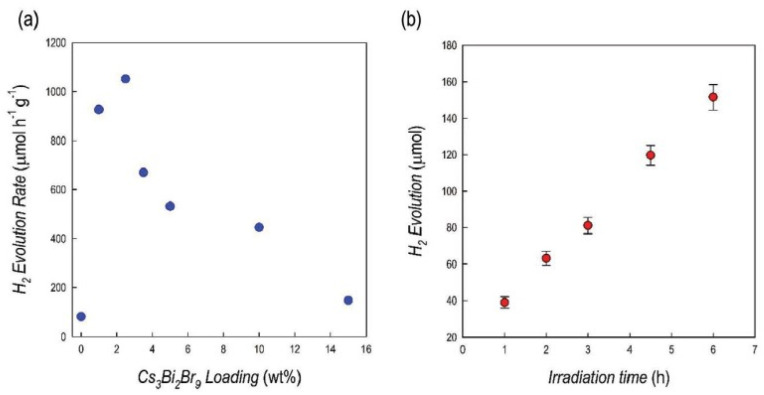
(**a**) Hydrogen evolution rates for Cs_3_Bi_2_Br_9_/g-C_3_N_4_ composites (1 g L^−1^) at different percentages of MHP loading in 10% *v*/*v* TEOA aqueous solution with Pt 3 wt%, under simulated solar light. (**b**) Hydrogen evolution profile over irradiation time for the 2.5% wt% Cs_3_Bi_2_Br_9_/g-C_3_N_4_ composites, under simulated solar light [35].

The solar-driven catalytic efficiency of the prepared composites has been determined in terms of HER by employing usual protocols reported from the literature, in 10% *v*/*v* aqueous triethanolamine (TEOA), as a typical sacrificial agent and with Pt (3% wt) as metal cocatalyst. In Figure 7a, they clearly showed the impressive enhancement of the HER from pure g-C_3_N_4_ (81µmol g^−1^ h^−1^) to 1% and 2.5% Cs_3_Bi_2_Br_9_/g-C_3_N_4_ composites, reaching the 2.5% loading, the highest HER of about ≈1050 µmol g^−1^ h^−1^ and also, in Figure 7b they reported the kinetics of H_2_ of the optimal 2.5% composite, indicating a linear increase of the hydrogen production as a function of time. The authors underlined that the pure Cs_3_Bi_2_Br_9_, under the same experimental condition, generated ≈22 µmol g^−1^ h^−1^ of H_2_. Further perovskite loading in the composites (>2.5%) leads to a progressive reduction of the efficiency of hydrogen photogeneration. This outstanding result clearly indicates a strong and effective synergy between the two semiconductors in the composites.

**Figure 8:**
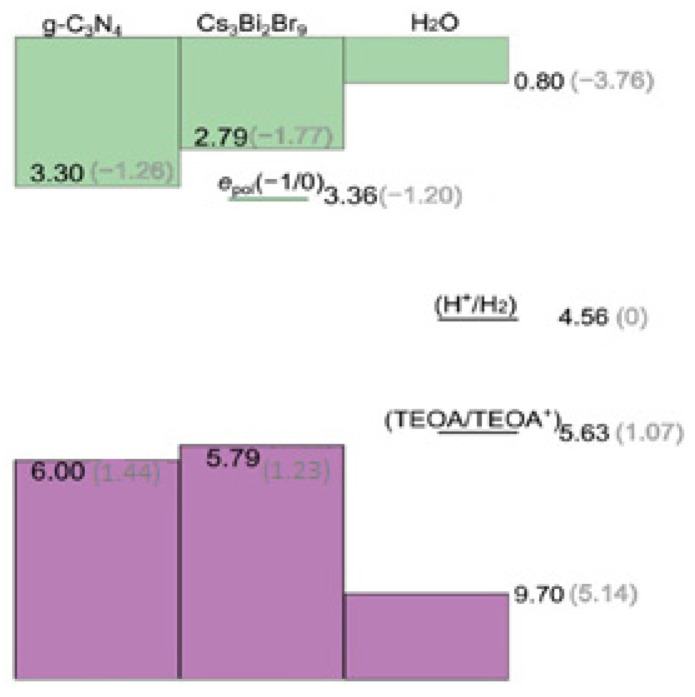
Valence band (VB) and conduction band (CB) edges of g-C_3_N_4_ and Cs_3_Bi_2_Br_9_ aligned with the band edges of liquid water and with the H^+^/H_2_ and TEOA/TEOA^+^ redox level through the vacuum level [3]. The (−1/0) charge transition level of the electron polaron calculated for Cs_3_Bi_2_Br_9_ is also reported. Values are referred to the vacuum level (black) and to the standard hydrogen electrode (SHE, grey) using the computational alignment achieved in ref (Y. Fu, H. Zhu, 2019) [36].

The alignment of the band edges between the constituents of the composite, shown in Figure 8, should boost the efficient charge separation; therefore, photogenerated holes in the valence band (VB) of g-C_3_N_4_ may migrate to that of Cs_3_Bi_2_Br_9_ while, in turn, photoexcited electrons in Cs_3_Bi_2_Br_9_ are transferred to the conduction band (CB) of g-C_3_N_4_. The efficient separation and transport of photoinduced electrons and holes induced by such an advantageous alignment of energy levels may hinder the bimolecular recombination of the charge carriers, thus enhancing the photocatalytic activity of the composite with respect to the separated materials.

This work, in addition to the remarkable results, further consolidated the promising route toward the application of lead-free MHPs as active photocatalytic materials for the realization of efficient heterojunctions with the application for H_2_ photo-evolution in an aqueous medium [36].

In the first part of this mini-review, we reported some approaches that have been explored in order to overcome the problematic presence of the toxic lead, with a special focus on 0D and 2D hybrid Bi-based perovskites, as well as the intrinsic instability of the majority of hybrid metal halide perovskite [13,14,15,37]. In the present section, we gave an account of the most appealing achievement regarding photocatalytic H_2_ generation exploiting full inorganic perovskites, and we will continue the section presenting another class of inorganic perovskite, the double perovskite that, as already mentioned above, is easily obtained by the substitution of two toxic lead ions with one monovalent and one trivalent less toxic metal ions. The 3D double perovskites, which crystal structure is shown in Figure 1, are a singular exception for Bi^3+^ ions because, as already reported, for this specific electronic configuration is more common the formation of low dimensional configurations [37]. The double perovskites present A_2_B(I)B(III)X_6_ stoichiometry, a modification of the classic 3D ABX_3_ perovskite, with [BX_6_]^4−^ octahedra that share all the corners, while the A^+^ cations are filling the spaces created by the alternated octahedra [4,6]. 

**Figure 9:**
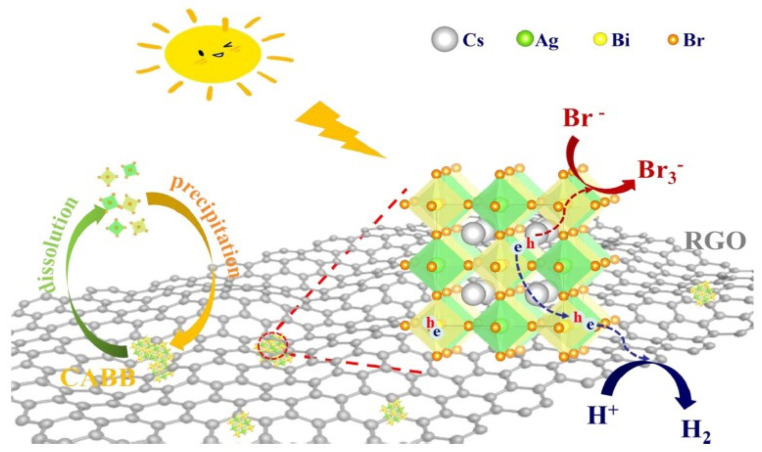
Schematic mechanism of H_2_ evolution by CABB/RGO under visible light irradiation. Reprinted with permission from ref [38]: Copyright 2019, Elvisier B.V. [38].

In 2020, Wang et al. reported, for the first time, the application of Cs_2_AgBiBr_6_ (CABB) natural lead-free double perovskite for hydrobromic (HBr) splitting under visible light irradiation. The reduced graphene oxide (RGO) cocatalyst, already employed in heterojunctions realization, with good electron transfer properties and acid stability, was introduced here to further improve the H_2_ production [38]. The photocatalytic activities of the CABB-based catalyst for H_2_ evolution were evaluated in aqueous saturated HBr, and H_3_PO_2_ solutions under visible light irradiation (λ ≥ 420 nm) and, in Figure 9, the authors reported the proposed mechanism for CABB/2.5% RGO photocatalytic H_2_ evolution. The photo-deposition of Pt on CABB provided slight improvement; on the other hand, when RGO was applied as a co-catalyst, the production of H_2_ increased greatly and reached 489 µmol g^−1^ when using the optimized CABB/2.5% RGO sample, which is 80 and 51 times higher than that of CABB and CABB/2.5% Pt, respectively [38]. To confirm the superior activity of CABB/2.5% RGO composites, 5 mg of GO was photo-reduced and tested for H_2_ evolution. The amount of hydrogen using 2.5% RGO alone was only 7.8% of CABB/2.5% RGO, further demonstrating the synergistic effect between CABB and RGO. The authors noticed that the content of RGO in CABB/xRGO composites and the concentration of H_2_PO_3_ in saturated solution have a great influence on HBr splitting. In particular, CABB/2.5% RGO showed extraordinary stability in a saturated solution, maintaining its high activity under 120 h of continuous photocatalytic H_2_ evolution. In conclusion, they proved that RGO accelerated the separation of photo-generated excitons and the electron transfer from CABB to RGO, thus reducing charge recombination and improving H_2_ production. These remarkable results opened the route for the application of naturally lead-free double perovskite as a promising candidate for heterojunctions construction for photocatalytic application [38]. 

**Figure 10:**
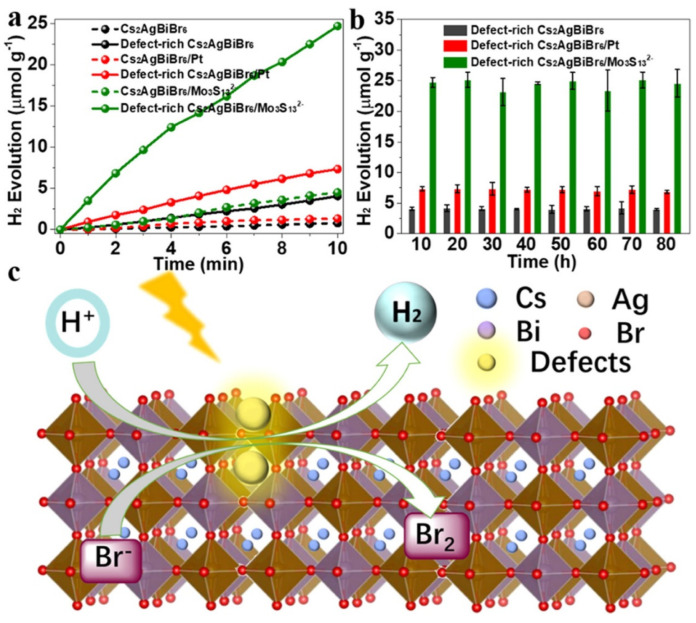
H_2_ evolution activity under visible light irradiation (**a**). Photocatalytic stability of the defect-rich Cs_2_AgBiBr_6_, Cs_2_AgBiBr_6_/Pt, Cs_2_AgBiBr_6_/Mo_3_S_13_^2−^ (**b**). Schematic illustration of H_2_ evolution over the defect-rich Cs_2_AgBiBr_6_ (**c**). Reprinted with permission from ref [39]: Copyright 2021, American Chemical Society [39].

In 2021, He et al. demonstrated the application of a modified Cs_2_AgBiBr_6_ perovskite for photocatalytic hydrogen evolution. They reported a post-synthesis visible light irradiation of Cs_2_AgBiBr_6_ to form a defect-rich surface showing that the formation of surface defects can promote surface charge separation by tuning the local atomic arrangement and electronic structure, leading to an important boosting in the photocatalytic efficiency of the defect-rich Cs_2_AgBiBr_6_. The authors explored different strategies to improve the poor efficiency and stability of the double perovskites and showed that the introduction of surface defects in Bi-based perovskite materials can both improve the photocatalytic performance by enhancing surface charge separation and decrease the number of coordinated active sites, which is favorable for numerous photocatalytic reactions [39]. With the enhancement of charge separation, the photocatalytic performance of H_2_ generation is increased by 5.27, 5.51, and 5.48 times relative to bulk Cs_2_AgBiBr_6_, Cs_2_AgBiBr_6_/Pt, Cs_2_AgBiBr_6_/Mo_3_S_13_^2−^, respectively. In addition, the as-prepared surface defective samples exhibited strong stability with no performance decrease even after 80h of activity [39]. These results are clearly shown in Figure 10, where it is possible to observe (10a) the amount of H_2_ evolved from the differently prepared samples, and more importantly, from 10b, it is clear that the defect-rich Cs_2_AgBiBr_6_ presents strong stability with no performance decrease even after an 80 h photocatalytic reaction.

The formation of the defect-rich surface was carried out by simple irradiation of the perovskite solution with a 300 W Xe-lamp fitted with a 420 nm cut-off filter. Cs_2_AgBiBr_6,_ after irradiation treatment, showed no additional diffraction peaks, confirming a high phase purity of the obtained sample, a result further corroborated by SEM and TEM measurements. The H_2_ generation of the defect-rich Cs_2_AgBiBr_6_ was investigated by exploiting a light intensity of 332.5 mW cm^−2^ with a visible light irradiation λ > 420 nm and evolved 4.06 µmol g^−1^ of H_2_ within 10 h, much higher compared with the amount generated by the pristine Cs_2_AgBiBr_6_ (0.77 µmol g^−1^). However, once the photocatalyst was deposited together with Pt or Mo_3_S_13_^2−^ nanoclusters as co-catalyst, the photocatalyst activity of the defect-rich Cs_2_AgBiBr_6_/Pt and defect-rich Cs_2_AgBiBr_6_/Mo_3_S_13_^2−^ increased up to 7.33 and 24.7 µmol g^−1^. Therefore, as shown in Figure 8c, the induction of surface defects can greatly improve the photocatalytic activity and stability of Cs_2_AgBiBr_6_ [39].

Following a similar path to Wang et al., in 2022, Jiang et al. presented an interesting way to enhance the photocatalytic performance of Cs_2_AgBiBr_6_ by forming composites materials supported on nitrogen-doped carbon (N-C) through a facile one-pot method. As we already mentioned, there are several semiconductors active in visible-light, which can be used in heterojunction formation. Here, the authors showed how nitrogen-carbon (N-C) materials can be a suitable candidate for efficient H_2_ evolution. They used N-C as cocatalyst and substrate for the in-situ one-step growth of Cs_2_AgBiBr_6_ to obtain Cs_2_AgBiBr_6_/N-C composites (Figure 9), which was then used for H_2_ evolution in HBr-saturated solution. They found that the optimal system, namely Cs_2_AgBiBr_6_/N-C-140 (140 label represents the hydrothermal process temperature), exhibited the best photocatalytic activity compared to pristine Cs_2_AgBiBr_6_ nanoparticles and the conventional physical mixture of Cs_2_AgBiBr_6_ and C-N-140. The HER of Cs_2_AgBiBr_6_/C-N-140 was 380 µmol g^−1^ h^−1^ under visible light irradiation (λ ≥ 420 nm, 300 W Xe lamp), dispersing 0.01 g of the amount of photocatalyst in a solution mixture containing 10 mL of saturated aqueous HBr and 2 mL of H_3_PO_2_, showing satisfactory stability for six cycles of photocatalytic H_2_ evolution [40].

**Figure 11:**
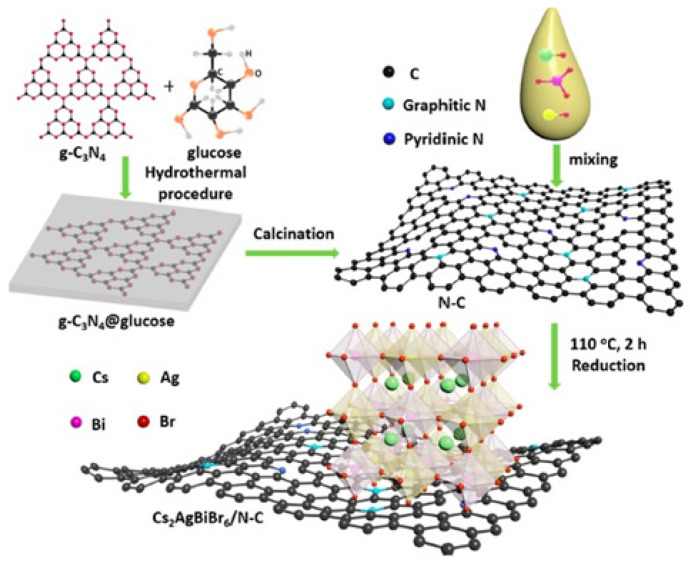
Preparation procedure of Cs_2_AgBiBr_6_/N-C photocatalyst. Reprinted with permission from ref [40]: Copyright 2021, American Chemical Society [40].

The key passage that represents the great difference between the physical mixture of the two catalysts and the one-pot in situ formation of the heterojunction consists of the dispersion of N-C substrates into aqueous HBr solution under ultrasonication, followed by mixing specific amounts of Cs_2_AgBiBr_6_ precursors and heating the mixture to 110 °C for 2 h (Figure 11).

The morphology and the structure of the obtained samples were characterized by TEM and SEM, and the Cs_2_AgBiBr_6_/N-C-140 composite showed a wrinkled and folded morphology compared to bare Cs_2_AgBiBr_6_ presenting a smooth surface, which may have a positive impact on the photocatalytic performance. Finally, the energy band study of the Cs_2_AgBiBr_6_/N-C-T_h_ composites showed how the CB of Cs_2_AgBiBr_6_ (−4.26 eV) is higher than the Fermi level of N-C-T_h_, and as a consequence, the photoexcited electrons could be transferred from the CB of Cs_2_AgBiBr_6_ to N-C-T_h_. The photoinduced electron transfer process results in the effective separation of electrons and holes, which effectively hinders electron-hole recombination and prolongs electron lifetime for efficient HBr reduction. The photocatalytic performances of Cs_2_AgBiBr_6_/N-C-T_h_ composites with N-C prepared at different hydrothermal temperatures were measured without the addition of any noble metals, and the performance of Cs_2_AgBiBr_6_/N-C-T_h_ decreased with increasing hydrothermal process temperature, from 140 °C to 200 °C. Moreover, the photoactivity of the Cs_2_AgBiBr_6_/N-C-140 composite increased with increasing the N-C-140 content from 2 to 5 wt%. It is interesting to note that Cs_2_AgBiBr_6_/ N-C-140 exhibits a better performance with respect to the system supported on Pt (Cs_2_AgBiBr_6_/Pt) and that the photocatalytic performance severely dropped without the addition of H_3_PO_2_, thus confirming the stabilizing role of H_3_PO_2_ to complete the photocatalytic process. The reported study represents a promising and general strategy to design and explore novel perovskite composites for H_2_ evolution [40].

To conclude this section, a very recent work of Huang et al., reported the successful synthesis, for the first time, of the NiCoP/Cs_2_AgBiBr_6_ (NCP/CABB) composite via electrostatic coupling [41]. Transition metal phosphides have shown great utility in the field of electrocatalysis due to their outstanding electron transfer ability, and since materials are good cocatalysts, they could represent a good alternative to noble metals for the promotion of electron transfer and electron-hole pair separation. In particular, NiCoP has recently attracted significant interest thanks to its outstanding electrochemical performance. The loading process of NCP on the surface of CABB broadened the visible light absorption range and, under visible light (λ ≥ 420 nm) irradiation, the NCP/CABB composite displayed excellent photocatalytic cycling stability, and HER of the 12.5% NCP/CABB composite reached 373.16 µmol g^−1^ h^−1^, exactly 88 times higher than the pristine CABB. Based on the extensive measurements and analysis, the authors reported the potential mechanism of photocatalytic H_2_ production, showing how, under visible light, electrons and holes were readily activated and jumped from VB of CABB to its CB, and, once the photogenerated electron-hole pairs were separated, most of the electrons from CABB were rapidly transferred to the CB of NCP promoting an efficient H_2_ generation reaction. The improvement of the performance of this system was ascribed to a load of NCP nanoparticles, which dramatically enhanced the visible light absorption and, at the same time, the excellent electron transport ability of NCP allowed an effective charge separation and transfer between CABB and NCP. This work demonstrated the feasibility and broad application prospects of the synergistic effect realized between Pb-free perovskites and transition metal phosphides [41].

## 4. Conclusions and Future Perspectives

In this Mini-Review, we reported state-of-the-art applications of Bi-based metal halide perovskites as photocatalysts in the hydrogen evolution reaction under visible light. Table 1 summarizes all the various systems described in this work, considering the environment medium, the sacrificial electron donor, and the amount of H_2_ produced. In addition to hydrogen generation reaction, Bi-based metal halide perovskite and derivatives have further shown promising applications in several photocatalytic reactions such as CO_2_ reduction, pollutant and dye degradation, and novel photochemical organic synthesis [19,41,42,43,44,45,46,47,48,49,50,51]. From the examples reported above, perovskite instability in aqueous media is still a major concern, and most of the results refer to hydrohalic acid splitting, with some exceptions for the full inorganic heterojunction, like g-C_3_N_4_/Cs_3_Bi_2_Br_9_ that proved water stability, and an important H_2_ amount production, suggesting that the type II heterojunction together with Z-scheme are the most promising configurations for H_2_ production, according to the literature [24,51]. Moreover, some examples of hybrid materials with enhanced water stability are starting to accumulate in the literature, where different chemical strategies are explored to afford this superior stability which allows direct hydrogen production from water. This aspect represents an important breaking point for future research in the field of perovskite-mediated photocatalysis. In this context, the high structural and composition variability represents a powerful tool to exploit the design of further perovskite and perovskite derivatives with improved water stability. Future research should also focus on the development of novel and improved heterojunctions between Bi-based MHPs and other semiconductors and/or metal nanoparticles to boost photocatalytic activity. Although much important progress has been achieved, there are still routes to be explored for further improvement of the photocatalytic performance, such as engineering the Bi-based materials to enhance their optical properties and stability; constructing more efficient heterojunctions, thus exploring other partners for the perovskites; and considering in more detail the construction of suitable electrode. It is clear that the very preliminary results discussed in this work laid the basis for the realization of an entirely novel class of photocatalyst materials with the potential to play an essential role in the energetic transition and reach an eco-sustainable carbon-neutral energy production society [3,4,6].

## Figures and Tables

**Table 1:** Summary of the photocatalytic Bi-based halide perovskite systems discussed in this review.

System	Source Power	Reaction Environment and Sacrificial Electron Donor	H_2_ (µmol g^−1^ h^−1^)	Ref.
**[DMA]_3_[BiI_6_]/PtI_x_** **Pt 1 wt%**	300 W Xe lamp (λ = 465 nm) for 100 h	H_3_PO_2_, HI sacrificial electron donor (1:4)	186.5	[30]
**MA_3_Bi_2_I_9_/Pt**	300 W Xe lamp (λ ≥ 420 nm) for 10 h	H_3_PO_2_, HI sacrificial electron donor, MABI saturated solution	169.2	[31]
**DMA_3_BiI_6_/MA_3_Bi_2_I_9_**	300 W Xe lamp (λ ≥ 420 nm) for 10 h	HI saturated solution, sacrificial electron donor	198	[32]
**(EtbtBi_2_I_10_)/Pt** **TiO_2_- EtbtBi2I10/Pt** **TiO_2_- EtbtBi_2_I_10_-rGO/Pt**	300 W Xe lamp (λ ≥ 420 nm) for 10 h	HI sacrificial electron donor, H_3_PO_2_ saturated solution	9.2 59.9 83.8	[33]
**Cs_3_Bi_0.6_Sb_1.4_I_9/_Pt**	300 W Xe lamp (λ ≥ 420 nm) for 10 h	HI sacrificial electron donor, CBI saturated solution and Cs_2_CO_3_	926	[35]
**DMA_3_BiI_6_/Pt**	Commercial LED for 6 h 425 nm, light intensity 8 mW	HI sacrificial electron donor, DAI solution	5.7	[34]
**g-C_3_N_4_/Cs_3_Bi_2_Br_9_ 2.5% wt%/Pt 3 wt%**	Simulated solar light 500 Wm 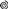 2 for 6 h UV filter	Aqueous solution with 10% *v*/*v* TEOA sacrificial electron donor	1050	[36]
**Cs_2_AgBiBr_6_/2.5%RGO**	300 W Xe lamp (λ ≥ 420 nm) for 3 h	HBr sacrificial electron donor, H_3_PO_2_ saturated solution	489	[38]
**Defect-rich** **Cs_2_AgBiBr_6_/Mo_3_S_13_^2−^** **Cs_2_AgBiBr_6_Defect-rich Cs_2_AgBiBr_6_** **Defect-rich Cs_2_AgBiBr_6_/Pt**	300 W Xe lamp (λ ≥ 420 nm) for 3 h	CABB saturated solution	24.7 0.8 4.1 7.3	[39]
**Cs_2_AgBiBr_6_/N-C-140**	300 W Xe lamp (λ ≥ 420 nm) for 3 h	HBr sacrificial electron donor, H_3_PO_2_ saturated solution	380	[40]
**Cs_2_AgBiBr_6_/NiCoP**	300 W Xe lamp (λ ≥ 420 nm) for 3 h	HBr sacrificial electron donor, H_3_PO_2_ saturated solution	373.2	[41]

## References

[1] Fu Y., Zhu H., Chen J., Hautzinger M.P., Zhu X.-Y., Jin S. (2019). Metal halide perovskite nanostructures for optoelectronic applications and the study of physical properties. Nat. Rev. Mater..

[2] Roknuzzaman M., Zhang C., Ostrikov K., Du A., Wang H., Wang L., Tesfamichael T. (2019). Electronic and optical properties of lead-free hybrid double perovskites for photovoltaic and optoelectronic applications. Sci. Rep..

[3] Romani L., Malavasi L. (2020). Solar-Driven Hydrogen Generation by Metal Halide Perovskites: Materials, Approaches, and Mechanistic View. ACS Omega.

[4] Armenise V., Colella S., Fracassi F., Listorti A. (2021). Lead-free metal halide perovskites for hydrogen evolution from aqueous solutions. Nanomaterials.

[5] Li J., Duan J., Yang X., Duan Y., Yang P., Tang Q. (2021). Review on recent progress of lead-free halide perovskites in optoelectronic applications. Nano Energy.

[6] Luo J., Zhang W., Yang H., Fan Q., Xiong F., Liu S., Li D.-S., Liu B. (2021). Halide perovskite composites for photocatalysis: A mini review. EcoMat.

[7] Stanley J.C., Mayr F., Gagliardi A. (2020). Machine Learning Stability and Bandgaps of Lead-Free Perovskites for Photovoltaics. Adv. Theory Simul..

[8] Corti M., Bonomi S., Chiara R., Romani L., Quadrelli P., Malavasi L. (2021). Application of metal halide perovskites as photocatalysts in organic reactions. Inorganics.

[9] Stroyuk O. (2018). Lead-free hybrid perovskites for photovoltaics. Beilstein J. Nanotechnol..

[10] Gao P., Grätzel M., Nazeeruddin M.K. (2014). Organohalide lead perovskites for photovoltaic applications. Energy Environ. Sci..

[11] Noel N.K., Stranks S.D., Abate A., Wehrenfennig C., Guarnera S., Haghighirad A.-A., Sadhanala A., Eperon G.E., Pathak S.K., Johnston M.B. (2014). Lead-free organic-inorganic tin halide perovskites for photovoltaic applications. Energy Environ. Sci..

[12] Mosconi E., Amat A., Nazeeruddin M.K., Grätzel M., de Angelis F. (2013). First-principles modeling of mixed halide organometal perovskites for photovoltaic applications. J. Phys. Chem. C.

[13] Hoefler S.F., Trimmel G., Rath T. (2017). Progress on lead-free metal halide perovskites for photovoltaic applications: A review. Monatsh. Chem..

[14] Ju M.G., Dai J., Ma L., Zeng X.C. (2017). Lead-Free Mixed Tin and Germanium Perovskites for Photovoltaic Application. J. Am. Chem. Soc..

[15] Shi Z., Guo J., Chen Y., Li Q., Pan Y., Zhang H., Xia Y., Huang W. (2017). Lead-Free Organic–Inorganic Hybrid Perovskites for Photovoltaic Applications: Recent Advances and Perspectives. Adv. Mater..

[16] Wani A.L., Ara A., Usmani J.A. (2015). Lead toxicity: A review. Interdiscip. Toxicol..

[17] Li X., Hoffman J.M., Kanatzidis M.G. (2021). The 2D halide perovskite rulebook: How the spacer influences everything from the structure to optoelectronic device efficiency. Chem. Rev..

[18] Tang Y., Mak C.H., Kai K.-C.C.-J., Meng F., Niu W., Li F.-F., Shen H.-H., Zhu X., Chen H.M., Hsu H.-Y. (2022). Lead-free hybrid perovskite photocatalysts: Surface engineering, charge-carrier behaviors, and solar-driven applications. J. Mater. Chem. A.

[19] Wu D., Zhao X., Huang Y., Lai J., Yang J., Tian C., He P., Huang Q., Tang X. (2021). Synthesis and CO2Photoreduction of Lead-Free Cesium Bismuth Halide Perovskite Nanocrystals. J. Phys. Chem. C.

[20] Abdin Z., Zafaranloo A., Rafiee A., Mérida W., Lipiński W., Khalilpour K.R. (2020). Hydrogen as an energy vector. Renew. Sustain. Energy Rev..

[21] Kim D., Lee D.K., Kim S.M., Park W., Sim U. (2020). Photoelectrochemical water splitting reaction system based on metal-organic halide perovskites. Materials.

[22] Kosco J., Bidwell M., Cha H., Martin T., Howells C., Sachs M., Anjum D., Lopez S.G., Zou L., Wadsworth A. (2020). Enhanced photocatalytic hydrogen evolution from organic semiconductor heterojunction nanoparticles. Nat. Mater..

[23] Maeda K., Domen K. (2016). Development of novel photocatalyst and cocatalyst materials for water splitting under visible light. Bull. Chem. Soc. Jpn..

[24] Tao R., Sun Z., Li F., Fang W., Xu L. (2019). Achieving Organic Metal Halide Perovskite into a Conventional Photoelectrode: Outstanding Stability in Aqueous Solution and High-Efficient Photoelectrochemical Water Splitting. ACS Appl. Energy Mater..

[25] Bosso P., Milella A., Barucca G., Mengucci P., Armenise V., Fanelli F., Giannuzzi R., Maiorano V., Fracassi F. (2021). Plasma-assisted deposition of iron oxide thin films for photoelectrochemical water splitting. Plasma Process. Polym..

[26] Guerrero A., Bisquert J. (2017). Perovskite semiconductors for photoelectrochemical water splitting applications. Curr. Opin. Electrochem..

[27] Zhai P., Haussener S., Ager J., Sathre R., Walczak K., Greenblatta J., McKone T. (2013). Net primary energy balance of a solar-driven photoelectrochemical water-splitting device. Energy Environ. Sci..

[28] Linsebigler A.L., Lu G., Yates J.T. (1995). Photocatalysis on Ti02 Surfaces: Principles, Mechanisms, and Selected Results. Chem. Rev..

[29] Low J., Yu J., Jaroniec M., Wageh S., Al-Ghamdi A.A. (2017). Heterojunction Photocatalysts. Adv. Mater..

[30] Zhao H., Li Y., Zhang B., Xu T., Wang C. (2018). PtIx/[(CH3)2NH2]3[BiI6] as a well-dispersed photocatalyst for hydrogen production in hydroiodic acid. Nano Energy.

[31] Guo Y., Liu G., Li Z., Lou Y., Chen J., Zhao Y. (2019). Stable Lead-Free (CH3NH3)3Bi2I9 Perovskite for Photocatalytic Hydrogen Generation. ACS Sustain. Chem. Eng..

[32] Tang Y., Mak C.H., Liu R., Wang Z., Ji L., Song H., Tan C., Barrière F., Hsu H.-Y. (2020). In Situ Formation of Bismuth-Based Perovskite Heterostructures for High-Performance Cocatalyst-Free Photocatalytic Hydrogen Evolution. Adv. Funct. Mater..

[33] Liu G.N., Zhao R.-Y., Xu B., Sun Y., Jiang X.-M., Hu X., Li C. (2020). Design, Synthesis, and Photocatalytic Application of Moisture-Stable Hybrid Lead-Free Perovskite. ACS Appl. Mater. Interfaces.

[34] Zhao H., Chordiya K., Leukkunen P., Popov A., Kahaly M.U., Kordas K., Ojala S. (2021). Dimethylammonium iodide stabilized bismuth halide perovskite photocatalyst for hydrogen evolution. Nano Res..

[35] Chen G., Wang P., Wu Y., Zhang Q., Wu Q., Wang Z., Zheng Z., Liu Y., Dai Y., Huang B. (2020). Lead-Free Halide Perovskite Cs3Bi2xSb2–2xI9 (x ≈ 0.3) Possessing the Photocatalytic Activity for Hydrogen Evolution Comparable to that of (CH3NH3)PbI3. Adv. Mater..

[36] Romani L., Speltini A., Dibenedetto C.N., Listorti A., Ambrosio F., Mosconi E., Simbula A., Saba M., Profumo A., Quadrelli P. (2021). Experimental Strategy and Mechanistic View to Boost the Photocatalytic Activity of Cs3Bi2Br9 Lead-Free Perovskite Derivative by g-C3N4 Composite Engineering. Adv. Funct. Mater..

[37] Igbari F., Wang Z.K., Liao L.S. (2019). Progress of Lead-Free Halide Double Perovskites. Adv. Energy Mater..

[38] Wang T., Yue D., Li X., Zhao Y. (2020). Lead-free double perovskite Cs2AgBiBr6/RGO composite for efficient visible light photocatalytic H2 evolution. Appl. Catal. B.

[39] He Z., Tang Q., Liu X., Yan X., Li K., Yue D. (2021). Lead-Free Cs2AgBiBr6Perovskite with Enriched Surface Defects for Efficient Photocatalytic Hydrogen Evolution. Energy Fuels.

[40] Jiang Y., Li K., Wu X., Zhu M., Zhang H., Zhang K., Wang Y., Loh K.P., Shi Y., Xu Q.-H. (2021). In Situ Synthesis of Lead-Free Halide Perovskite Cs2AgBiBr6Supported on Nitrogen-Doped Carbon for Efficient Hydrogen Evolution in Aqueous HBr Solution. ACS Appl. Mater. Interfaces.

[41] Huang Q., Guo Y., Chen J., Lou Y., Zhao Y. (2022). NiCoP modified lead-free double perovskite Cs2AgBiBr6 for efficient photocatalytic hydrogen generation. New J. Chem..

[42] Dai Y., Tüysüz H. (2019). Lead-Free Cs3Bi2Br9 Perovskite as Photocatalyst for Ring-Opening Reactions of Epoxides. ChemSusChem.

[43] Bresolin B.M., Sgarbossa P., Bahnemann D.W., Sillanpää M. (2020). Cs3Bi2I9/g-C3N4 as a new binary photocatalyst for efficient visible-light photocatalytic processes. Sep. Purif. Technol..

[44] Estrada-Pomares J., Ramos-Terrón S., Lasarte-Aragonés G., Lucena R., Cárdenas S., Rodríguez-Padrón D., Luque R., de Miguel G. (2022). Mechanochemically designed bismuth-based halide perovskites for efficient photocatalytic oxidation of vanillyl alcohol. J. Mater. Chem. A Mater..

[45] Shi M., Zhou H., Tian W., Yang B., Yang S., Han K., Li R., Li C. (2021). Lead-free B-site bimetallic perovskite photocatalyst for efficient benzylic C–H bond activation. Cell Rep. Phys. Sci..

[46] Zhang Z., Wang B., Zhao H.-B., Liao J.-F., Zhou Z.-C., Liu T., He B., Wei Q., Chen S., Chen H.-Y. (2022). Self-assembled lead-free double perovskite-MXene heterostructure with efficient charge separation for photocatalytic CO2 reduction. Appl. Catal. B.

[47] Liu Z., Yang H., Wang J., Yuan Y., Hills-Kimball K., Cai T., Wang P., Tang A., Chen O. (2021). Synthesis of lead-free Cs2AgBix6 (X = Cl, Br, I) double perovskite nanoplatelets and their application in CO2 photocatalytic reduction. Nano Lett..

[48] Wu D., Zhao X., Huang Y., Lai J., Li H., Yang J., Tian C., He P., Huang Q., Tang X. (2021). Lead-free perovskite Cs2AgBiX6nanocrystals with a band gap funnel structure for photocatalytic CO2reduction under visible light. Chem. Mater..

[49] Zhou L., Xu Y.F., Chen B.X., Kuang D.B., Su C.Y. (2018). Synthesis and Photocatalytic Application of Stable Lead-Free Cs2AgBiBr6 Perovskite Nanocrystals. Small.

[50] Sun Q., Ye W., Wei J., Li L., Wang J., He J.-H., Lu J.-M. (2022). Lead-free perovskite Cs3Bi2Br9 heterojunctions for highly efficient and selective photocatalysis under mild conditions. J. Alloys. Compd..

[51] Xiao Z., Song Z., Yan Y. (2019). From Lead Halide Perovskites to Lead-Free Metal Halide Perovskites and Perovskite Derivatives. Adv. Mater..

